# Standardized distances for placement of REBOA in patients with aortic stenosis

**DOI:** 10.1038/s41598-020-70364-9

**Published:** 2020-08-07

**Authors:** Markus Harboe Olsen, Tasalak Thonghong, Lars Søndergaard, Kirsten Møller

**Affiliations:** 1 Department of Neuroanaesthesiology, Rigshospitalet, University of Copenhagen, Copenhagen, Denmark; 2 Department of Cardiology, Rigshospitalet, University of Copenhagen, Copenhagen, Denmark

**Keywords:** Interventional cardiology, Cardiac device therapy

## Abstract

Resuscitative endovascular balloon occlusion of the aorta (REBOA) is a technique where a balloon is advanced through the common femoral artery and temporarily inflated for treatment of cardiac arrest or non-compressible haemorrhage. The aim of this study was to measure intravascular distances relevant for correct placement of the REBOA catheter using computer tomographic (CT) scans. In a series of CT scans of the aorta from 100 patients diagnosed with severe aortic stenosis planned for transcatheter aortic valve implantation, we measured the intravascular distance from the insertion site in the common femoral artery to two potential zones for placement of the REBOA catheter; between the left subclavian artery and the celiac trunk (Zone 1), as well as between the aortic bifurcation and the distal take-off of the renal arteries (Zone 3). The mean (± SD) intravascular distance from the femoral artery to intra-aortic Zone 1 was 36 (± 2.5) cm for the lower border and 60 (± 4.1) cm for the upper border, respectively. For intra-aortic Zone 3, the mean (± SD) intravascular distance was 21 (± 2.1) cm to the lower border and 31 (± 2.3) cm to the upper border. Calculated potentially safe intervals for placement of the REBOA in Zone 1 was with 99.7% likelihood between 43 and 48 cm. No similar potentially safe interval could be calculated for Zone 3. According to this cohort study of patients with severe aortic stenosis, the balloon of the REBOA catheter should travel intraarterially between 43 (lower limit) and 48 cm (upper limit) from the site of insertion into the common femoral artery, which would lead to correct placement in intra-aortic Zone 1 in 99.7% of cases. In contrast, no potential safety interval could be similarly defined for insertion in Zone 3.

## Introduction

Until the introduction of resuscitative endovascular balloon occlusion of the aorta (REBOA), compression of the descending or abdominal aorta was done with external clamping via resuscitative thoracotomy or laparotomy during severe haemorrhage to minimize blood loss^1,2^. Occlusion of the aorta minimizes the haemorrhage from arteries distal to the occlusion and simultaneously redirects the circulating blood to upper body, e.g. the coronary and cerebral vascular territories^3^. Complete occlusion of the aortic lumen is possible for several minutes without complications, which may allow for surgical repair of the injury^4–6^, or for cardio-pulmonary resuscitation to achieve recovery of spontaneous circulation in the case of cardiac arrest.

**Figure 1:**
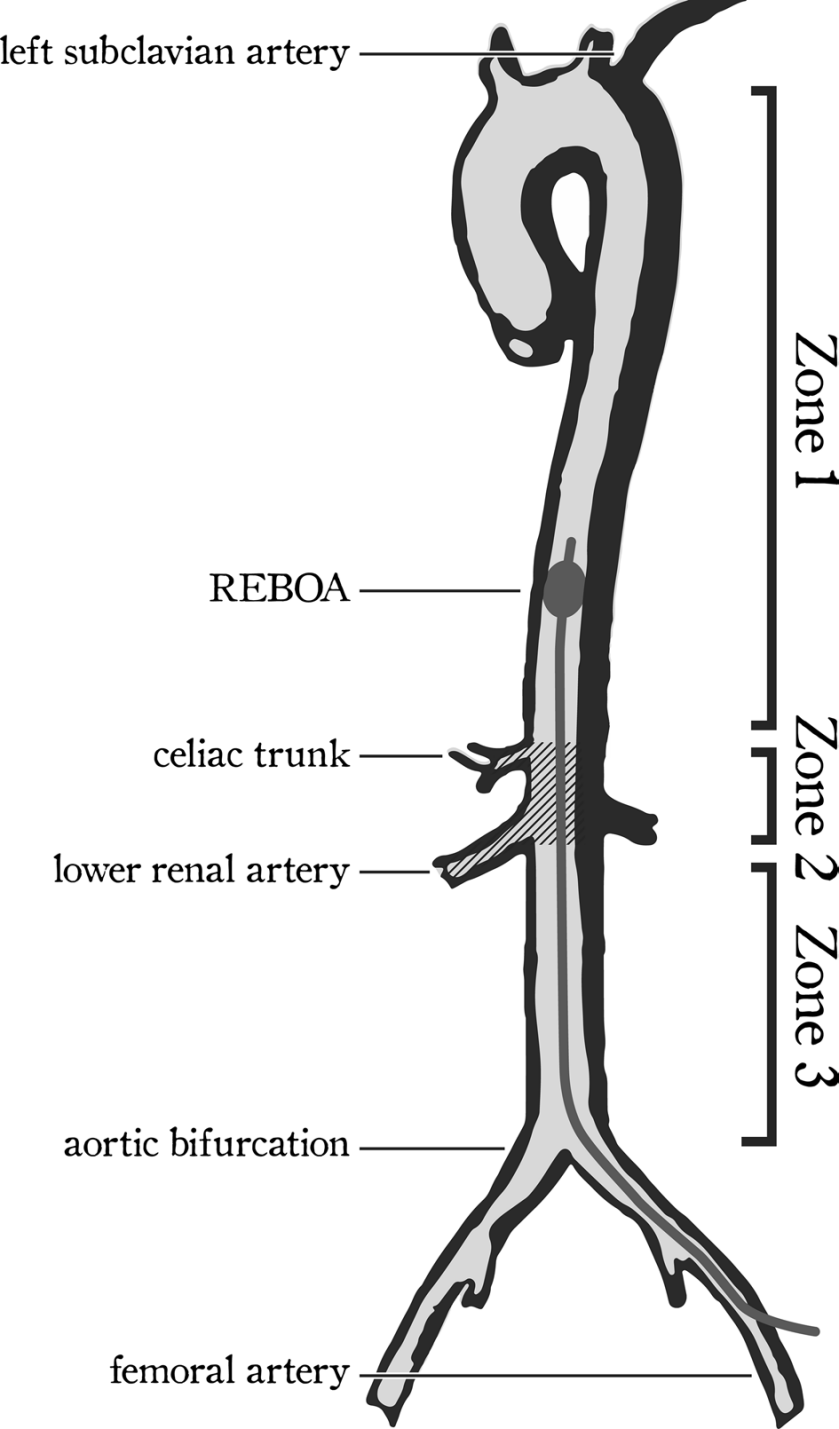
Simplistic rendering of aorta. Zone 1 (from left subclavian artery to the upper border of the celiac trunk), Zone 2 (the upper border of the celiac trunk to the lower border of the distal take-off of the renal arteries), and Zone 3 (from the lower border of the lower renal artery to the aortic bifurcation). Zone 1 is occluded in the case of cardiac arrest or life-threatening intra-abdominal hemorrhage; Zone 2 has no current indication; and Zone 3 is occluded in the case of life-threatening pelvic or lower limb haemorrhage^7^. *REBOA* Resuscitative Endovascular Balloon Occlusion of the Aorta.

Depending on the type of injury, the REBOA catheter is inserted intra-arterially through the common femoral artery and a balloon is inflated when it is located between the celiac trunk and the left subclavian artery (Zone 1), or between the aortic bifurcation and the distal border of the renal arteries (Zone 3). In contrast, occlusion between the celiac trunk and the distal border of the renal artery (Zone 2) is generally not recommended (the so-called no-occlusion zone) (Fig. 1)^7^. The indications for REBOA include, but are not limited to, intraabdominal and pelvic hemorrhage, as well as traumatic and non-traumatic cardiac arrest^8^.

Inflation with the balloon located in Zone 1 is used for resuscitation after non-compressible torso hemorrhage or after cardiac arrest. In this case, the length of the REBOA catheter should allow balloon placement above the celiac trunk to stop perfusion of the lower trunk and extremities but must be placed below the lower limit of the left subclavian artery to protect cerebral perfusion. Conversely, Zone 3 occlusion is used for uncontrollable pelvic and lower-extremity haemorrhage, which cannot be controlled by tourniquets; for this indication, the balloon should be located above the aortic bifurcation and below the lower renal artery.

A major current limitation in the use of the REBOA device is the need of radiographic guidance for safe placement, which considerably limits its prehospital or bedside usage^2^. The available literature on REBOA catheter placement without radiographic guidance^9–12^ includes both successes and failures; thus an effective non-radiography-based REBOA method has yet to be developed^13–15^. In the meantime, unguided placement of REBOA carries a risk of incorrect positioning of the occluding balloon, potentially leading to balloon rupture, pressure damage, non-perfusion of critical organs or unintended perfusion of non-critical organs or hemorrhaging arteries. Length markers and external measurement of the patient torso have both been investigated as methods to determine zone placement of the REBOA catheter. However, further simplification of the setup may conceivably increase the success rate, decrease the time to catheter insertion and successful balloon inflation^14,15^ and optimize the feasibility of REBOA treatment in the prehospital or bedside setting.

One such simplification is to calculate a standardized insertion length of the REBOA catheter that leads to correct placement of the balloon in the desired intra-aortic zone with an acceptable success rate. The first step would be to investigate the intravascular distances from the hypothetical insertion point to anatomical landmarks that define the borders of these zones in a relevant patient population.

The aim of this study was to test the hypothesis that standardized insertion lengths of a REBOA catheter balloon may ensure inflation in intra-aortic Zone 1 or 3, respectively, without radiographic guidance and irrespective of the height of individual patients. To this end, we measured intravascular distances from a well-defined entry point in the left common femoral artery to anatomical landmarks and calculated confidence intervals for the distances that would ensure correct location inside intra-aortic Zones 1 and 3, respectively. Previously only trauma patients have been studied^16,17^, and with the recent proposal to include of REBOA in treatment of non-traumatic cardiac arrest this study is done in an elderly population.

## Materials and methods

A series of computer tomographic scans (CT) from randomly selected patients who had previously been diagnosed with severe, symptomatic aortic stenosis and planned for transcatheter aortic valve implantation (TAVI) were retrospectively evaluated. All scans had been conducted between June 2017 and April 2018 and were selected from the archive at the Department of Cardiology, Rigshospitalet, Denmark (Table 1).

**Table 1:** Characteristics of the study population.

	All (n = 100)			Men (n = 62)			Women (n = 38)			*P* value*
	Mean	SD	Range	Mean	SD	Range	Mean	SD	Range	
Age (years)	78	8	42;89	76	9	42;88	78	6	66;89	< 0.05
Height (cm)	170	10	140;193	175	7	162;193	162	8	140;178	< 0.0001
BMI (^kg^/_m_^2^)	26.5	5.1	17.0;44.7	27.4	5.3	19.1;44.7	25.0	4.42	17;42	< 0.05

*SD* standard deviation, *BMI* body mass index.

*Student’s *t* test for unpaired samples, men versus women.

The insertion site for the balloon catheter was defined as a point in the left femoral artery 3 cm below the mid-inguinal point, i.e. the midpoint between the anterior superior iliac spine and pubic tubercle. Aortic zones were defined as follows: Zone 1: the zone extending from the upper border of the celiac trunk to the lower border of the left subclavian artery; Zone 2, from the lower border of the lowest of the renal arteries to the upper border of the celiac trunk; and Zone 3, from the aortic bifurcation to the lower border of the lowest of the renal arteries (Fig. 1)^7^.

The intravascular distance from the baseline to the following anatomical landmarks was measured: the aortic bifurcation; the lower border of the lowest of the renal arteries; the lower border of the celiac trunk; the center of the celiac trunk; the upper border of the celiac trunk; the level of the diaphragm; and the lower border of the left subclavian artery. All distances were measured, by one clinician (TT), in the midline of the femoral, external and common iliac arteries and the aorta. All measurements were made in 3MENSIO (Maastricht, The Netherlands, 2018) by a specialist cardiologist at the Department of Cardiology, Rigshospitalet, Denmark.

With the use of available scans and data the Regional Committee in the Capital Region of Denmark on Health Research Ethics (Protocol number: 17024428; September 8, 2017) ruled that according to Danish law no approval or consent from patients were necessary. The study was furthermore approved by the Director of the Department of Cardiology, Rigshospitalet, Denmark, and carried out in accordance with regional guidelines.

### Statistical analysis

Analyses were performed using R (R 4.0.0, R Core Team [2020], Vienna, Austria)^18^. All data were normally distributed as asserted by histograms and the Shapiro–Wilk test. Quantitative data were given as mean (SD).

Continuous variables were compared between groups using Student’s *t* test for unpaired data. For measurement of anatomical distances, the mean as well as 95% (2 SD) and 99.7% (3 SD) confidence limits were calculated for potentially safe placement of a REBOA catheter balloon inside intra-aortic zones. This allowed us to determine the 95% and 99.7% intervals of potentially safe placement of the REBOA balloon in Zone 1 and 3 as follows:

In Zone 1, the potentially safe interval was located between the upper confidence limit of the distance to the upper border of the celiac trunk (the lower limit of Zone 1) and the lower confidence limit of the distance to the lower border of the subclavian artery (the upper limit);

In Zone 3, the potentially safety interval was located between the upper confidence limit of the distance to the aortic bifurcation (the lower limit of the Zone 3) and the lower confidence limit of the distance to the lower border of the most distal renal artery (the upper limit).

Linear regression was used to measure an association between distances to anatomical landmarks and height, weight, age and sex. *P* values of < 0.05 were considered significant.

### Ethics approval and consent to participate

With the use of available scans and data the Regional Committee on Health Research Ethics (Protocol number: 17024428) ruled that according to Danish law no approval was necessary. The study was furthermore approved by the Director of the Department of Cardiology, Rigshospitalet, Denmark.

## Results

One hundred patients with aortic valve stenosis (62 males and 38 females) were included (Table 1). Distances from the common femoral artery to anatomical landmarks are given in Table 2. Sex and height were significantly associated with the distances to the landmarks that defined the borders of both Zone 1 and Zone 3 (Supplemental Data A).

**Table 2:** Distance from baseline to anatomical locations (cm).

	All (n = 100)			Men (n = 62)			Women (n = 38)		
	Mean	SD	Range	Mean	SD	Range	Mean	SD	Range
B to distal to left subclavian artery	60	4.1	50;69	61	4	52;69	58	3.5	50;65
B to diaphragm	39	3.2	32;49	39	3.2	32;49	39	3.2	34;45
B to proximal to celiac trunk	36	2.5	30;42	37	2.3	32;42	35	2.5	30;40
B to celiac trunk	36	2.5	31;44	36	2.2	31;41	35	2.5	30;40
B to distal to celiac trunk	35	2.5	29;41	36	2.2	31;41	34	2.5	29;40
B to distal to renal artery	31	2.3	25;37	32	2.1	27;37	30	2.1	25;35
B to aortic bifurcation	21	2.1	16;28	22	2.2	17;28	21	1.8	16;25
B to middle of Zone 1^a^ (calc.)	48	3.1	41;55	49	2.9	42;55	47	2.9	40;53
B to middle of Zone 2^b^ (calc.)	34	2.3	28;40	34	2.1	29;40	32	2.1	28;38
B to middle of Zone 3^c^ (calc.)	26	2.0	21;32	27	2	23;32	25	1.8	21;30

*B* baseline, *SD* standard deviation, *Calc.* calculated positions.

^a^Between below left subclavian artery and above celiac trunk.

^b^Between below lowest renal artery and above celiac trunk.

^c^Between below lowest renal artery and aortic bifurcation.

**Figure 2:**
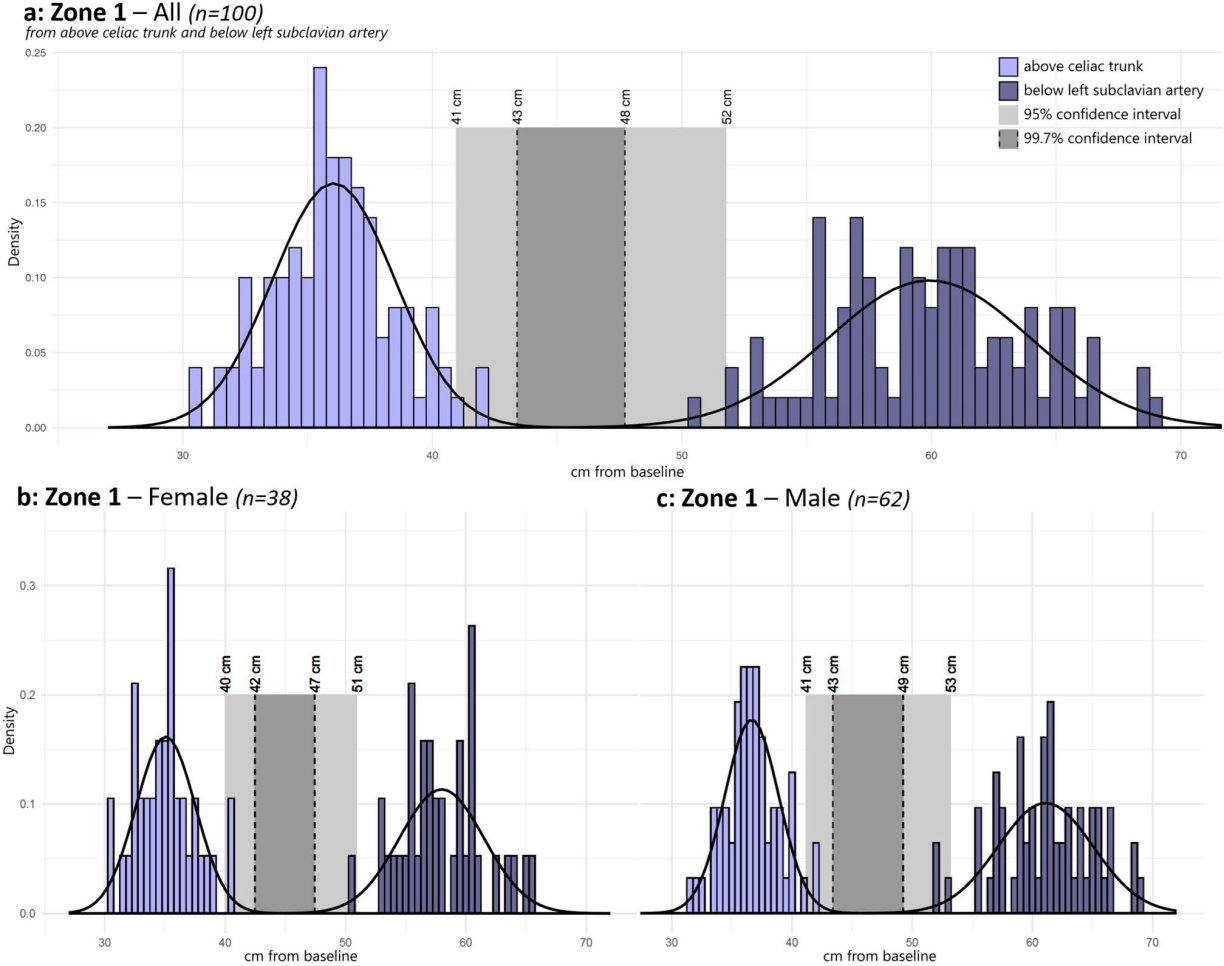
Location of Zone 1 in cm from the femoral arterial baseline in all (**a**), in female (**b**) and in male (**c**). The upper border (below left subclavian artery) and lower border (above celiac trunk) of Zone 1 were plotted into a histogram with grouping data in blocks of 0.5 cm and above a calculated normal distribution curve. Light grey rectangle defines the 95% confidence interval for placement in Zone 1. Dark grey rectangle defines the 99.7% confidence interval for placement in Zone 1.

The 99.7% safety interval for the intra-aortic Zone 1 was 43 to 48 cm, whereas the 95% safety interval was 41 to 52 cm. As expected, no patients in the cohort had anatomical distances that would lead to incorrect placement of the REBOA catheter balloon, if it was placed inside the 99.7% safety interval in Zone 1. In contrast, using the 95% safety interval, four patients in this cohort would have experienced incorrect balloon placement (Fig. 2a). Similar subgroup analyses resulted in only a marginal change in the safety intervals between male and female (Fig. 2b,c).

**Figure 3:**
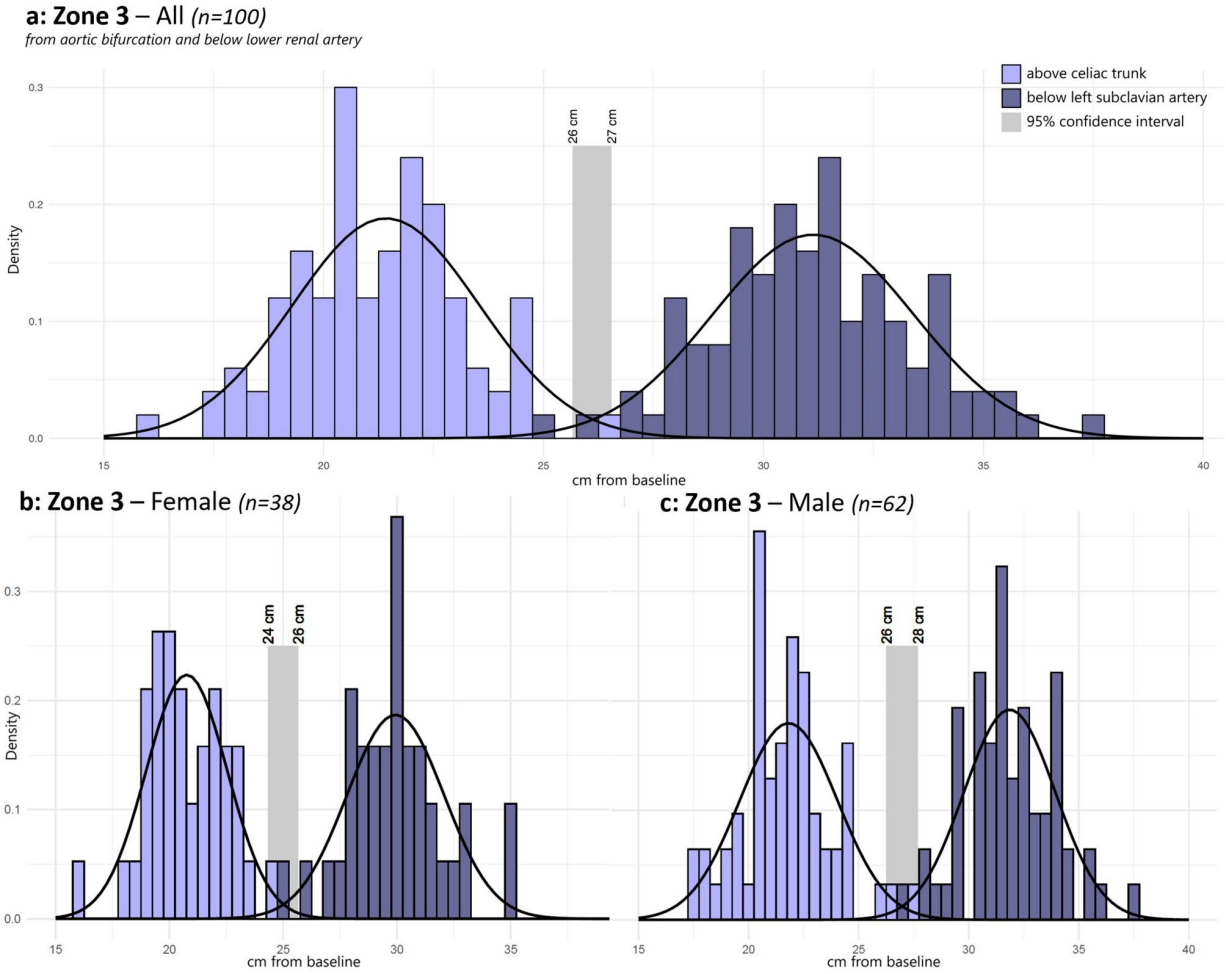
Location of Zone 3 in cm from the femoral arterial baseline in all (**a**), in female (**b**) and in male (**c**). The upper border (below lower renal artery) and lower border (at aortic bifurcation) of Zone 1 were plotted into a histogram with grouping data in blocks of 0.5 cm and above a calculated normal distribution curve. Light grey rectangle defines the 95% confidence interval for placement in Zone 3. No 99.7% confidence interval could be defined for placement in Zone 3.

For Zone 3, the 99.7% safety interval of could not be defined, because the 99.7% confidence limits of the upper and lower border overlapped each other. However, a 95% safety interval was calculated as 26 to 27 cm; in the present cohort, six patients would have received incorrectly placed balloon, if this safety interval had been used (Fig. 3a). The subgroup analyses showed the 95% safety interval varied between genders (Fig. 3b+c).

## Discussion

To ensure a minimal risk of wrong placement of REBOA, a 99.7% confidence point was preferable even though a 95% confidence interval is normally acceptable. With this in mind, this study suggests a relatively safe interval for placement of the REBOA catheter balloon in Zone 1 between 43 and 48 cm from the hypothetical baseline in the femoral artery. In contrast, an equally safe interval could not be defined in Zone 3.

The upper limit of Zone 1 (48 cm) found in our study was somewhat higher than the limits reported in three previous publications investigating the placement of REBOA^16,19,20^ (Supplemental Data B), but corresponds with a large study investigating REBOA placement in a large trauma population^17^. This suggests that the optimal placement should be in the lower part of Zone 1, to minimize the risk of misplacement, also when adding gender-related differences into the equation. Additionally, linear regression suggested a relationship between height and the safety limits of the zone. Thus, our findings may not apply to populations with different distributions of height.

Zone 3 is shorter than Zone 1, and we were unable to identify a similar potentially safe interval to place the REBOA device in Zone 3. With 95% certainty placement could be placed between 26 and 27 cm, and this increased risk would have to be balanced with the potential life-saving benefits. This was in agreement with Pezy et al.^16^ who also found overlapping confidence intervals. Thus, a standardized length cannot currently be recommended for Zone 3. In the case of uncontrollable pelvic hemorrhage, one of the possible indications for occlusion of Zone 3, in which REBOA might be lifesaving, prehospital occlusion in Zone 1 could be a possible solution, until radiographic guidance is possible.

Prehospital or bedside REBOA might also help a large group of patients with non-hypovolemic and non-traumatic cardiac arrest as a mean to increase central perfusion. Even though standard cardiopulmonary resuscitation may sustain a cardiac output equal to the one seen in heart failure^21^, this is insufficient to obtain or retain a shockable rhythm for many patients^22^.

Patients surviving cardiac arrest have a high risk of brain injury^14,23^. REBOA may redirect the blood flow to the heart and brain and thereby increase both post-cardiac arrest survival and neurological outcome. To expand the use of REBOA, not only a standardized length, but experienced providers are needed to ensure safe and fast insertion of a REBOA catheter.

The finding that safe REBOA balloon placement in Zone 1 is limited to patients resembling those studied in this and previous cohorts^16,19^. Pezy et al.^16^ also found an association with ethnicity, which further underlines the necessity for caution in extrapolation. Finally, measuring the length from baseline to an anatomical landmark with CT images does not consider the actual intra-arterial behavior of a REBOA catheter; thus, the actual intra-arterial length of a catheter might deviate from an externally measured length. The measured length is estimated by a line through the center of the lumen following the course of the aorta. A catheter might not follow this exact route, especially in patients with a tortuous aorta^24^.

Notwithstanding these limitations, the identification of a safe interval for REBOA placement is a first step to implement safe prehospital or bedside REBOA.

## Conclusion

Placement of REBOA into Zone 1 using a standardized insertion length may be possible and safe in a population with demographics resembling the present study. Reconciling the present results with previous studies, we recommend targeting the lower part of Zone 1 to minimize the risk of incorrect placement.

Finally, we could not identify a similar safety interval for placement of the REBOA in Zone 3 due to overlapping intervals for the upper and lower anatomical borders of this zone.

## Supplementary information

Supplementary Information.

## Data Availability

The datasets generated and analysed during the current study are not publicly available due to Danish Law but are available from the corresponding author on reasonable request and only after signing a Data Processing Agreement.
