# Supplementary material for: Standardized distances for placement of REBOA in patients with aortic stenosis
Source: Sci Rep. 2020 Aug 7;10:13410. doi: 10.1038/s41598-020-70364-9 (PMC7414869; doi:10.1038/s41598-020-70364-9)
Supplement: Supplementary file 1 — Supplementary Information. [file 41598_2020_70364_MOESM1_ESM.docx]

# Supplemental Data A – Univariable linear regression model for each anatomic location

|  | **Sex** | **Age** (numeric) | **Height** (numeric) | **Weight** (numeric) |
| --- | --- | --- | --- | --- |
| B to distal to left subclavian artery | 1.39 (1.18-1.68)* | 0.96 (0.64-1.44) | 3.24 (2.09-5.02)* | 2.31 (0.99-5.39) |
| B to Diaphragm | 1.07 (0.94-1.22) | 0.85 (0.51-1.43) | 1.14 (0.61-2.14) | 0.72 (0.24-2.14) |
| B to proximal to celiac trunk | 1.35 (1.12-1.66)* | 0.66 (0.34-1.28) | 4.27 (1.97-9.24)* | 2.35 (0.57-9.75) |
| B to Celiac trunk | 0.92 (0.78-1.08) | 0.39 (0.21-0.73)* | 1.05 (0.47-2.35) | 1.66 (0.41-6.66) |
| B to distal to celiac trunk | 1.35 (1.12-1.66)* | 0.69 (0.35-1.34) | 4.21 (1.94-9.12)* | 2.06 (0.50-8.59) |
| B to distal to renal artery | 1.58 (1.26-2.04)* | 0.68 (0.33-1.40) | 6.55 (2.95-14.6)* | 4.41 (0.98-19.8) |
| B to Aortic bifurcation | 1.30 (1.06-1.65)* | 0.45 (0.21-0.96)* | 5.45 (2.24-13.3)* | 4.88 (0.96-24.8) |

Data is presented as odds ratio (95% confidence interval). B: Baseline; * Significant p value < 0.05.

# Supplemental Data B – Measurements from similar studies

|  | *Okada et al.* ^19^ | | | *Pezy et al.* ^16^ | | | *Linnebur et al.* ^20^ | | |
| --- | --- | --- | --- | --- | --- | --- | --- | --- | --- |
| n | 25 | | | 280 | | | 10 | | |
|  | mean | (SD) | [range] | mean | (SD) | [range] | mean^a^ | (SD)^a^ | [range]^a^ |
| Age | 50 | (20.7) | [21;83] | 38.8 | (16.5) | [16;90] | 64.2 | (10.7) | [43;75] |
| Height (cm) | 164 | (7.12) | [145;177] | 171 | (8.7) | [150;198] | 168 | (10.1) | [152;183] |
| BMI | 22.9 | (2.33) | [15.5;27.8] | 24.0 | (4.5) | [15.6;45.2] | 19.4 | (3.1) | [13.3;23.4] |
| B – left subclavian artery | 56.5 | (2.8) | [52;62] | 54.6 | (2.9) | [47;65] | 54.7 | (3.3)^b^ | - |
| B – celiac trunk | 32.0 | (1.6) | [29;36] | 32.6 | (1.7) | [27;41] | 32.9 | (2.2)^b^ | - |
| B – lower renal artery | - | - | - | 29.4 | (1.7) | [25;37] | - | - | - |
| B – aortic bifurcation | - | - | - | 19.7 | (1.7) | [15;28] | - | - | - |

^a^ Calculated from data in article. ^b^ n = 9.
